# Supplementary material for: From sequence to enzyme mechanism using multi-label machine learning
Source: BMC Bioinformatics. 2014 May 19;15:150. doi: 10.1186/1471-2105-15-150 (PMC4229970; doi:10.1186/1471-2105-15-150)
Supplement: Additional file 2 — Java code of ml2db. Additional file ml2db_code.tar.gz contains the Java source code to run the multi-label machine learning experiments and save the results to database. The code’s Javadoc is included. [file 1471-2105-15-150-S2.zip › additional file 2/ml2db/ml2db/doc/main/ml2db/weka/DataSetWriter.html]

DataSetWriter


JavaScript is disabled on your browser.


- Overview
- Package
- Class
- Use
- Tree
- Deprecated
- Index
- Help

- Prev Class
- Next Class

- Frames
- No Frames

- All Classes

- Summary:
- Nested |
- Field |
- Constr |
- Method

- Detail:
- Field |
- Constr |
- Method


main.ml2db.weka

## Class DataSetWriter

- java.lang.Object
- - main.ml2db.weka.DataSetWriter

- ---

    

  ```
  public class DataSetWriter
  extends java.lang.Object
  ```

  Class

  Version:
  :   3 Sep 2010

  Author:
  :   Luna De Ferrari ldf(at)st-andrews.ac.uk

- - ### Constructor Summary

    Constructors

    Constructor and Description

    `DataSetWriter(DataSetManager manager)`
  - ### Method Summary

    Methods

    | Modifier and Type | Method and Description |
    | --- | --- |
    | `java.lang.String` | `attributesSection(weka.core.Instances instances)` Gets the weka arff string header including all attributes and the "@data" label, but excluding all instances. |
    | `java.lang.String` | `dataStatistics()` |
    | `DataSetManager` | `getDataSetManager()` |
    | `java.lang.String` | `getFilePath()` |
    | `java.lang.String` | `getLog()` |
    | `java.lang.String` | `logHeader()` |
    | `java.lang.String` | `logTail()` |
    | `void` | `write()` |
    | `void` | `writeAttributesAndClasses(weka.core.Instances instances)` Write the data instances to file |
    | `void` | `writeDataStatistics()` Logs to file the number of instances, classes and attributes in the dataset. |
    | `void` | `writeHeader()` |
    | `void` | `writeInstanceToFile(java.lang.String instanceName, weka.core.Instance instance, java.lang.String filePath)` Write an individual Weka instance to file |
    | `void` | `writeTail()` |

    - ### Methods inherited from class java.lang.Object

      `equals, getClass, hashCode, notify, notifyAll, toString, wait, wait, wait`

- - ### Constructor Detail


    - #### DataSetWriter

      ```
      public DataSetWriter(DataSetManager manager)
      ```
  - ### Method Detail


    - #### attributesSection

      ```
      public java.lang.String attributesSection(weka.core.Instances instances)
      ```

      Gets the weka arff string header including all attributes and the "@data"
      label,
      but excluding all instances.

      Parameters:
      :   `instances` -

      Returns:
      :   the weka arff string header including all attributes and the
          "@data" label, but excluding all instances.


    - #### dataStatistics

      ```
      public java.lang.String dataStatistics()
      ```


    - #### getDataSetManager

      ```
      public DataSetManager getDataSetManager()
      ```


    - #### getFilePath

      ```
      public java.lang.String getFilePath()
      ```


    - #### getLog

      ```
      public java.lang.String getLog()
      ```


    - #### logHeader

      ```
      public java.lang.String logHeader()
      ```


    - #### logTail

      ```
      public java.lang.String logTail()
      ```


    - #### write

      ```
      public void write()
      ```


    - #### writeAttributesAndClasses

      ```
      public void writeAttributesAndClasses(weka.core.Instances instances)
      ```

      Write the data instances to file


    - #### writeDataStatistics

      ```
      public void writeDataStatistics()
      ```

      Logs to file the number of instances, classes and attributes in the
      dataset.


    - #### writeHeader

      ```
      public void writeHeader()
      ```


    - #### writeInstanceToFile

      ```
      public void writeInstanceToFile(java.lang.String instanceName,
                             weka.core.Instance instance,
                             java.lang.String filePath)
      ```

      Write an individual Weka instance to file


    - #### writeTail

      ```
      public void writeTail()
      ```


- Overview
- Package
- Class
- Use
- Tree
- Deprecated
- Index
- Help

- Prev Class
- Next Class

- Frames
- No Frames

- All Classes

- Summary:
- Nested |
- Field |
- Constr |
- Method

- Detail:
- Field |
- Constr |
- Method
